# Lysine-Specific Histone Demethylase 1A Regulates Macrophage Polarization and Checkpoint Molecules in the Tumor Microenvironment of Triple-Negative Breast Cancer

**DOI:** 10.3389/fimmu.2019.01351

**Published:** 2019-06-12

**Authors:** Abel H. Y. Tan, WenJuan Tu, Robert McCuaig, Kristine Hardy, Thomasina Donovan, Sofiya Tsimbalyuk, Jade K. Forwood, Sudha Rao

**Affiliations:** ^1^Epigenetics and Transcription Laboratory Melanie Swan Memorial Translational Centre, Sci-Tech, University of Canberra, Canberra, ACT, Australia; ^2^School of Biomedical Sciences, Charles Sturt University, Wagga Wagga, NSW, Australia

**Keywords:** macrophage polarization, LSD1, CoREST, breast cancer, epigenetics, tumor microenvironment, tumor associated macrophages

## Abstract

Macrophages play an important role in regulating the tumor microenvironment (TME). Here we show that classical (M1) macrophage polarization reduced expression of LSD1, nuclear REST corepressor 1 (CoREST), and the zinc finger protein SNAIL. The LSD1 inhibitor phenelzine targeted both the flavin adenine dinucleotide (FAD) and CoREST binding domains of LSD1, unlike the LSD1 inhibitor GSK2879552, which only targeted the FAD domain. Phenelzine treatment reduced nuclear demethylase activity and increased transcription and expression of M1-like signatures both *in vitro* and in a murine triple-negative breast cancer model. Overall, the LSD1 inhibitors phenelzine and GSK2879552 are useful tools for dissecting the contribution of LSD1 demethylase activity and the nuclear LSD1-CoREST complex to switching macrophage polarization programs. These findings suggest that inhibitors must have dual FAD and CoREST targeting abilities to successfully initiate or prime macrophages toward an anti-tumor M1-like phenotype in triple-negative breast cancer.

## Introduction

Breast cancer is the most common female cancer worldwide (1). The triple-negative subtype of breast cancer (TNBC) accounts for 15–20% of cases (2, 3) and is characterized by an absence of estrogen receptor (ER), progesterone receptor (PR), and human epidermal growth factor 2 (HER2) expression (3–5). TNBC patients have a worse prognosis than patients with other breast cancer subtypes, not least because they do not have the targets and so do not respond to hormonal or HER2-targeting therapies. Various novel treatments have been trialed in patients with TNBC, but standard chemotherapy regimens remain the standard of care (5–7).

TNBCs have a particularly high immune cell infiltrate compared to other breast cancer subtypes, but these immune cells are often functionally impaired (8, 9). The tumor microenvironment (TME) of any cancer contains a complex mixture of immune cells with both pro- and anti-tumor properties. Tumor-associated macrophages (TAMs) are a major immune cell subset in the TME, where they exist along a phenotypic spectrum from classically (M1) to alternatively (M2) activated (10, 11). Lipopolysaccharide (LPS) and IFN-γ-induced M1 macrophages secrete pro-inflammatory cytokines and reactive oxygen/nitrogen species that contribute to tumor cell cytotoxicity. Conversely, IL-4- and IL-13-induced M2 macrophages produce anti-inflammatory cytokines that can suppress other immune cells in the TME and promote tumor progression (12–14).

Epigenetic programming plays a significant role in regulating macrophage polarization and can be manipulated using various inhibitors (15). Numerous epigenetic enzymes control DNA methylation, histone methylation, and histone acetylation [see reviews in (16–18)]. Lysine-specific demethylase 1 (LSD1) is a H3K4 and H3K9 demethylase essential for myeloid cell differentiation (19), reactivating key immune checkpoint regulators, producing cytotoxic T cell chemokines (20), and preventing IL6 silencing in LPS-tolerant macrophages (21). We previously showed that immune-incompetent mice treated with the LSD1 inhibitor phenelzine had a higher proportion of M1-like macrophages in the TME of xenografts (22). We also showed that LSD1 is critical for reprogramming cancer stem cell (CSC)-inducible gene signatures and directly regulates distinct CSC genes implicated in breast cancer metastasis by tethering to their promoter regions (22).

Here we show that M1 (IFN-γ + LPS) or M2 (IL-4) macrophages differentially express LSD1 and nuclear serine 111 phosphorylated LSD1 (LSD1-s111p). LSD1 and LSD1-s111p downregulation in the M1 (IFN-γ + LPS) phenotype correlates with decreased nuclear activity and increased expression of histone H3 lysine 4 dimethylation (H3K4me2) and histone H3 lysine 9 dimethylation (H3K9me2) marks and decreased interactions with nuclear REST corepressor 1 (CoREST) and zinc finger protein SNAI1 (SNAIL) complexes. Phenelzine treatment mimics the phenotype of these M1 (IFN-γ + LPS) polarized cells by disrupting the LSD1-CoREST complex unlike the catalytic inhibitor GSK2879552. Thus, showing the importance of targeting the LSD1-CoREST complex to epigenetically prime macrophages toward an M1-like phenotype. *In vivo*, LSD1 inhibition by phenelzine primes TAMs to express M1-like gene that displayed both common and unique pathways to the chemotherapeutic protein-bound paclitaxel (Abraxane). Phenelzine treatment also led to a higher proportion of macrophages expressing M1 like protein (iNOS, CD86 and PD-L1) in formalin-fixed paraffin embedded (FFPE) tissue sections of tumors from a murine model of triple negative breast cancer (TNBC). Collectively, our data show for the first time that LSD1 inhibitors that target the LSD1 FAD and disrupt the LSD1-CoREST complex leading to a destabilization of LSD1 can epigenetically prime macrophages toward a M1-like phenotype in the TME, and future immunomodulatory drug development must take LSD1 FAD and LSD-CoREST complex into account to improve efficacy.

## Materials and Methods

### Cell Culture

RAW264.7 cells (ATCC TIB-71) were cultured in high-glucose DMEM with 2 mM L-glutamine, 1 x penicillin-streptomycin-neomycin (PSN) (Gibco, Thermo Fisher Scientific, Waltham, MA), and 10% heat-inactivated fetal calf serum (FCS). 4T1 cells (ATCC CRL-2539) were cultured in DMEM with 2 mM L-glutamine, PSN, and 10% heat-inactivated FBS. Transfection reactions were performed with 10 nM mouse LSD1 siRNA (sc-60971) and mock siRNA (sc-37007) (Santa Cruz Biotechnology Inc., Dallas, TX) using Lipofectamine 2,000 (Invitrogen, Carlsbad, CA).

### *In vitro* Macrophage Polarization

RAW264.7 cells were seeded into 6- or 12-well plates 24 h before polarizing macrophages. M1 (IFN-γ + LPS) classical activation was induced by adding 100 ng/ml lipopolysaccharide (LPS) and 20 ng/ml IFN-γ, and M2 (IL-4) alternative activation was induced by adding 20 ng/ml IL-4 for 24 h. Phenelzine and GSK2879552 (GSK) were added at 500 μM for 24 h.

### RNA Extraction and Quantitative Real-Time PCR

Total RNA was extracted from RAW264.7 cells using the RNeasy Micro kit (Qiagen, Hilden, Germany) according to the manufacturer's protocols. RNA was measured using the Nanodrop spectrophotometer (Thermo Fisher Scientific) and reverse transcribed into cDNA using the SuperScript VILO cDNA synthesis kit using the manufacturer's protocols. TaqMan quantitative real-time PCR was performed with the following mouse TaqMan probes: *Nos2* (Mm00440502_m1), *Gpr18* (Mm02620895_s1), *IL6* (Mm00446190_m1), *Fpr2* (Mm00484464_s1), *IL12b* (Mm00434174_m1), *ILb* (Mm00434228_m1), *CCR7* (Mm01301785_m1), *Myc* (Mm00487804_m1), *Egr2* (Mm00456650_m1), *Arg1* (Mm00475988_m1), *Mrc1* (Mm00485148_m1), *Mgl2* (Mm00460844_m1), *Pdcd1* (Mm01285676_m1), *CD274* (Mm03048248_m1), *Pdcd1lg2* (Mm00451734_m1), KDM1A (Mm01181029_m1), and *Gapdh* (Mm99999915_g1). DNA from formaldehyde-assisted isolation of regulatory elements (FAIRE) was quantified by SYBR real-time PCR with the primer set listed in Supplementary Table 1. qPCR data were normalized to *Gapdh* loading control.

### Formaldehyde-Assisted Isolation of Regulatory Elements (FAIRE)

FAIRE samples were prepared as outlined in Simon et al. (23). Briefly, cells were cross-linked with 1% formaldehyde and lysed. The cell lysates were sonicated to yield an average DNA fragment distribution of ~200–500 bp. A 50 μl aliquot of fragmented DNA (total input control DNA) was reverse cross-linked at 65°C followed by phenol-chloroform extraction. The remaining sonicated DNA (FAIRE DNA) was directly isolated by phenol-chloroform extraction and purified using the Zymo-SpinTM I kit (Zymo Research, Irvine, CA).

### Animal Studies

Five-week-old female BALB/c mice were obtained from the Animal Resources Center (ARC), Perth, and allowed to acclimatize for 1 week in the containment suites at The John Curtin School of Medical Research (JCSMR). All experimental procedures were performed in accordance with the guidelines and regulations approved by the Australian National University Animal Experimentation Ethics Committee (ANU AEEC). Mice were shaved at the site of inoculation the day before subcutaneous injection with 2 ×10^5^ 4T1 cells in 50 μl PBS into the right mammary gland. Treatment was started at day 12 post inoculation, when tumors reach approximately 50 mm^3^. Tumors were measured using external calipers and volumes calculated using a modified ellipsoidal formula $\frac{1}{2}$ (*a*/*b*^2^), where *a* = longest diameter and *b* = shortest diameter. Mice were treated with Abraxane (30 mg/kg) and PD1 (10 mg/kg) every 5 days (twice) and phenelzine (40 mg/kg) daily. All treatments were given intraperitoneally in PBS. Tumors were collected on day 27 post-inoculation of 4T1 cells for flow cytometry, macrophage enrichment for NanoString, and immunofluorescence microscopy.

### Tumor Dissociation Protocol

4T1 tumors were harvested in cold DMEM supplemented with 2.5% FCS before being finely cut using surgical scalpels and enzymatically dissociated using collagenase type 4 (Worthington Biochemical Corp. Lakewood, NJ) at a concentration of 1 mg collagenase / 1 g of tumor at 37°C for 1 h. Dissociated cells were then passed through a 0.2 μM filter before downstream assays.

### Flow Cytometry

Single cell suspensions were prepared as in the tumor dissociation protocol. Non-specific labeling was blocked using anti-CD16/32 (Fc block; BD Biosciences, Franklin Lakes, NJ) before specific labeling. BD Horizon fixable viability stain 780 was used to distinguish live and dead cells. Tumor cells were stained with antibodies targeting F4/80 PE, CD206 APC, and Ly6C Brilliant Violet 421 (all from BioLegend, San Diego, CA). Sample acquisition was performed with the BD LSR II cytometer and results analyzed with FlowJo software.

### Macrophage Enrichment and NanoString nCounter Protocol

Single cell suspensions were magnetically labeled with anti-F4/80 microbeads UltraPure (Miltenyi Biotec, Bergisch Gladbach, Germany) in MACS running buffer. Macrophages were then positively isolated using the autoMACS Pro Separator (Miltenyi Biotec, Bergisch Gladbach, Germany) according to the manufacturer's protocols. Enriched cells were then snap frozen and RNA isolated using the RNeasy Mini kit (Qiagen). Samples were analyzed using the NanoString platform according to the manufacturer's procedures. Briefly, 100 ng of RNA was hybridized with the mouse myeloid innate immunity panel codeset for 18 h at 65°C. Samples were then loaded onto the chip via the nCounter prep station and data acquired using the nCounter Digital Analyzer. Data analysis was performed using nSolver Analysis Software. The Benjamini-Yekutelli method was used to calculate the false discovery rate (FDR) (24).

### Immunofluorescence

Cells were cultured for 24 h on sterilized coverslips and then for a further 24 h after treatment with either complete medium, 100 ng/mL LPS and 20 ng/mL IFN-γ, 20 ng/mL IL-4, 500 μM phenelzine, or 500 μM GSK to form the treatment groups: control, M1 (IFN-γ + LPS), M2 (IL-4), phenelzine, and GSK, respectively.

After culturing, cells were fixed with 3.7% paraformaldehyde and permeabilized using 2% Triton X-100 solution. Cells were then blocked using 1% bovine serum albumin (BSA) and probed with rabbit-LSD1p (ABE1462, EMD Millipore, Burlington, MA), mouse-H3K9me2 (ab1220, Abcam, Cambridge, UK), goat-H3K4me2 (ab11946, Abcam), mouse-CD38 (102716, BioLegend), and goat-SNAIL1 (sc-10433, Santa Cruz Biotechnology) followed by visualization with corresponding secondary antibodies (all Thermo Fisher Scientific): anti-rabbit (A21206 and A10042), anti-mouse (A10037), and anti-goat (A21082 and A11055) conjugated to either Alexa Fluor 488, or 568, or 633. Coverslips were mounted onto glass microscope slides using SlowFade™ Diamond Antifade Mountant with DAPI.

Formalin-fixed, paraffin-embedded melanoma primary tumor biopsies were processed in the BondRX for OPAL staining (Perkin-Elmer, Waltham, MA) using the instrument protocol: ER1 for 20 min at 100°C with Epitope Retrieval Solution (pH6 Citrate-based retrieval solution) followed by probing with primary antibodies to F4/80 (ab100790, Abcam), iNOS (ab115819, Abcam), CD86 (ab213044, Abcam) and PD-L1 (ab2386097, Abcam) (for the M1 panel) or F4/80, EGR2 (ab90518, Abcam), CD206 (ab64693, Abcam) and PD-L2 (PAB12986, Abnova) (for the M2 panel). Primary antibodies were visualized with an Opal Kit 520, 570, 650, and 690. Coverslips were mounted on glass microscope slides with ProLong Clear Antifade reagent (Life Technologies, Carlsbad, CA). Opal kits used: 7-color automation kit (NEL801001KT) and the 4-color automation kit (NEL820001KT).

Slides were observed under a Leica DMi8 inverted microscope running Leica Application Suite X software. Multiple images were taken at various positions on the slide using a 100x oil immersion lens. Images were analyzed using ImageJ software, with the fluorescence intensity measured from a minimum of 20 cells and an average total fluorescence of either the nucleus or cytoplasm reported. Background fluorescence was measured and subtracted from all results.

For high-throughput microscopy, protein targets were localized by confocal laser scanning microscopy. Single 0.5 μm sections were obtained using an Olympus-ASI automated microscope with 100x oil immersion lens running ASI software. The final image was obtained by employing a high throughput automated stage with ASI spectral capture software. Digital images were analyzed using automated ASI software (Applied Spectral Imaging, Carlsbad, CA) to automatically determine the distribution and intensities with automatic thresholding and background correction of either the average nuclear fluorescent intensity (NFI) and average or whole cell total fluorescent intensity (TFI). The plot-profile feature of ImageJ was used to plot the fluorescence signal intensity along a single line spanning the nucleus (*n* = 5 lines per nucleus, 5 individual cells) using the average fluorescent signal intensity for the indicated pair of antibodies plotted for each point on the line with SE. Signal was plotted to compare how the signals for each antibody varied compared to the opposite antibody. For each plot-profile, the PCC was determined in ImageJ. PCC indicates the strength of relationship between the two fluorochrome signals for at least 20 individual cells ± SE. Colors from representative images correspond to plot-profiles.

### LSD1 Activity Assay

Nuclear extracts were prepared as previously described from cells, and 5 μg of protein/well in triplicate was used to measure LSD1 demethylase activity using the Abnova LSD1 Demethylase Activity/Inhibition assay kit (Abnova, Taipei City, Taiwan) according to the manufacturer's protocol.

### X-Ray Crystallography

Recombinant human LSD1 encoding residues 173-830 was expressed in *E. coli* BL21(DE3)pLysS using auto-induction medium (Studier, 2005) from the pMSCG21 expression vector. Cells were grown at 25°C, harvested by centrifugation, and resuspended in His buffer A (50 mM phosphate buffer, 300 M NaCl, and 20 mM imidazole). Resuspended cells were lysed by two repetitive freeze-thaw cycles and the cell lysate clarified by centrifugation. The soluble cell lysate was filtered using a 0.45 μm low protein binding filter and injected onto a 5 mL Ni-Sepharose HisTrap HP column equilibrated with His buffer A. Following sample injection, the column was washed with 15 column volumes of His buffer A, then eluted with His buffer A containing 500 mM imidazole. The purified protein was purified further by size exclusion chromatography and applied to a pre-equilibrated Superdex 200 26/60 size exclusion column. The protein was concentrated to 17 mg/ml using an Amicon ultracentrifugal device with a 10 kDa molecular weight cut off, aliquoted and stored at −80°C. The LSD1 protein was screened for conditions that induce crystals, with diffraction quality crystals obtained in 2–15% PEG 3350, ammonium citrate pH 6.5 and pH 7.0. A 10-molar excess of phenelzine sulfate or GSK2879552 was added to the LSD1 prior to crystallization, with a notable change in color from yellow to pale yellow/clear.

All X-ray diffraction data were collected on the MX1 crystallography beamline at the Australian Synchrotron. Images were indexed and integrated in iMosfilm (25), and data merged and scaled in Aimless (26). The number of molecules within the asymmetric unit was estimated based on the Matthews coefficient (VM) and the predicted molecular weight of the protein (27, 28). Model building and refinement was performed using COOT (29) and REFMAC (30).

### Bioinformatic Analysis

Promoter and enhancer analysis was performed on significant genes (*p* < 0.05, False Discovery Rate (FDR) < 0.15) up or down-regulated by phenelzine from the NanoString nCounter assay. Benjamini-Yekutieli false discovery rate method was used to calculate the FDR (24). Enhancer regions are from Supplementary Table 1 in Ostuni et al. (31). Raw data was downloaded from GEO, GSE38377, GSE91009 and GSE78873 and adapter trimmed and mapped to mm9 using Trimmomatic (32) and Bowtie2 (33) in Galaxy. CpG and GC, and histone levels were calculated with HOMER (34). Counts for promoters [were 1 kb ± around the transcription starts site (TSS)], and enhancer regions (using the given range). Accessibility and histone levels for stimulated and non-stimulated cells were equalized to the mean promoter values for all RefSeq genes. Bedtools (35) was used to detect which enhancers were within 10 kb of the gene TSS. Welch two sample *t*-test and boxplots were performed in R.

### Statistics

All statistical comparisons between sample groups were calculated using the two-tailed non-parametric Mann-Whitney test (GraphPad Prism, San Diego, CA) unless otherwise indicated. Where applicable, statistical significance is denoted by ^*^P ≤ 0.05, ^**^P ≤ 0.005, ^***^P ≤ 0.0005 and ^****^P ≤ 0.0001. Data are expressed as mean ± SE.

## Results

### Phenelzine and GSK2879552 Modify the LSD1 Flavin Adenine Dinucleotide (FAD) Cofactor

We previously showed that LSD1 modulates epithelial to mesenchymal transition in CSCs and that LSD1 inhibition promotes an M1-type response in an immune-deficient mouse cancer xenograft model (22). Here we aimed to further characterize the effect of LSD1 inhibition on macrophage polarization utilizing two different LSD1 inhibitors, phenelzine, and GSK2879552 (GSK).

**Figure 1:**
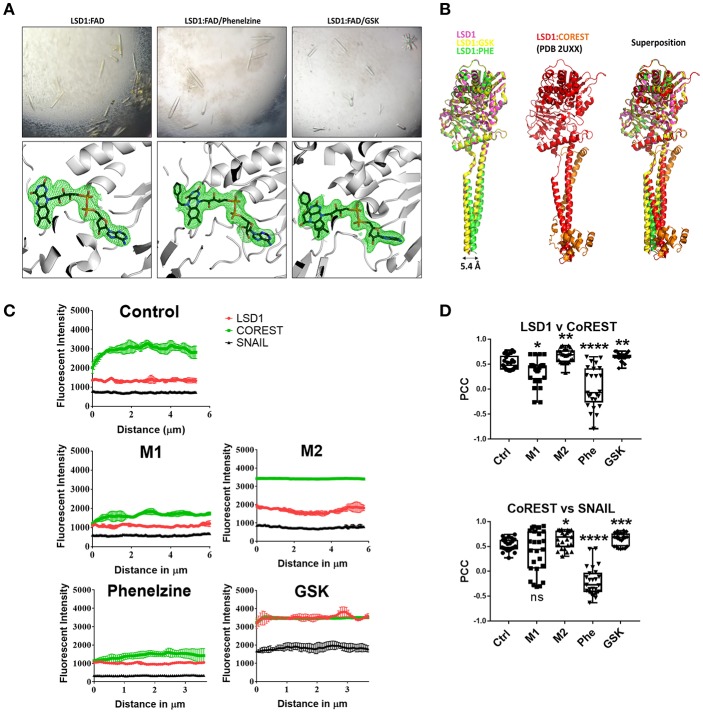
Phenelzine targets the FAD domain of LSD1 and potentially disrupts the LSD1/CoREST axis resulting in destabilization of LSD1 and its nuclear activity. **(A)** LSD1 protein crystals (top panel) grown in the absence (left) and presence of phenelzine (middle) and GSK2879552 (right). In the absence of inhibitors, strong density (bottom panels) was observed consistent with the FAD cofactor (shown as sticks colored with carbons black, nitrogen blue, oxygen red, and phosphate orange). The map is a simulated annealed omit map for FAD contoured at 2.5 sigma. LSD1 is colored gray and shown in cartoon mode. The FAD modifications by phenelzine (middle) and GSK (right) are supported by strong density, with corresponding maps and colors as per LSD1:FAD. **(B)** Structural superposition of LSD1 in the absence and presence of phenelzine and GSK. The structures solved in this study (left panel), LSD1 alone (pink) (PDB 6NQM), LSD1:GSK (yellow) (PDB 6NQU), and LSD1:phenelzine (green) (PDB 6NR5) are represented in cartoon mode. These structures are superimposed in the left panel, showing a high degree of structural homology in the LSD1 catalytic domain for all three structures. LSD1 alone and LSD1:GSK also show high structural conservation in the alpha-helical tails; however, LSD1:phenelzine has a 5.4 Å displacement in this region. This region is important for CoREST binding, as shown in the middle panel (PDB 2UXX). Superposition of all structures in the left and middle panels is shown on the right, highlighting that CoREST binding is mediated by the correct position of these domains. All images were generated in Pymol. **(C)** The plot-profile feature of ImageJ was used to plot the fluorescence signal intensity along a single line spanning the nucleus (*n* = 5 lines per nucleus, 5 individual cells) using the average fluorescent signal intensity for the indicated pair of antibodies plotted for each point on the line with SE. **(D)** Pearson's correlation coefficient (PCC) indicating the colocalization of LSD1/CoREST and CoREST/SNAIL. ^*^p < 0.05, ^**^p< 0.005, ^***^p < 0.0005, ^****^p < 0.0001 Mann–Whitney *t*-test.

Phenelzine and GSK are monoamine oxidases (MAOs) and effective LSD1 inhibitors (36, 37), probably via modification of the flavin adenine dinucleotide (FAD) cofactor (38, 39). We first confirmed whether the inhibitors act via a FAD-dependent mechanism by crystallizing LSD1 in the presence and absence of phenelzine and GSK. The gross crystal morphology was markedly different: yellow in the absence of inhibitor and translucent in the presence of phenelzine or GSK (Figure 1A). However, the crystals diffracted to similar resolution and belonged to the same space group (Supplementary Table 2), each containing one LSD1 molecule in the asymmetric unit.

In the absence of inhibitor, there was clear density corresponding to FAD (Figure 1A). In the presence of phenelzine, there was clear positive density at the central nitrogen of the flavin moiety on FAD, consistent with a previously determined structure of human MAO-B in the presence of phenelzine (PDB 2VRM). This is also consistent with the observed color change, since the flavin moiety confers these spectral properties (40). Similarly, LSD1 protein crystals grown in the presence of GSK exhibited clear additional positive density at the flavin moiety, consistent with a previous crystal structure of LSD1 bound to a GSK analog (PDB 2UXX) (41). Both inhibitors appear to act via similar mechanisms and modify the FAD cofactor at the flavin moiety.

The inhibitor-bound structures were superimposed and compared with the native LSD1 structure to examine whether these inhibitors resulted in any other observable structural changes. Phenelzine resulted in a small 5.4Å shift in the long alpha-helical tails of LSD1 (residues 415–514) (PDB 6NR5) that was not present in LSD1 (PDB 6NQM) or LSD1:GSK2879552 crystals (PDB 6NQU). This region mediates CoREST binding, making it possible that phenelzine-induced structural changes in this region may also affect LSD1 activity outside the catalytic region (Figure 1B).

### LSD1 Inhibitors Differentially Target or Disrupt the LSD1/CoREST Complex in Macrophages

Given that phenelzine-induced structural changes in the CoREST region (Figure 1B) may affect LSD1 activity outside the catalytic region, we examined the impact of phenelzine on the LSD1/CoREST complex and its impact on macrophage polarization by comparing unpolarized, M1 or M2 polarized, and phenelzine- or GSK-treated RAW264.7 cells *in vitro* by high-resolution fluorescent confocal microscopy (Figure 1C). Phenelzine treatment or M1 polarization with IFN-γ and LPS significantly reduced the number and nuclear expression of CoREST, LSD1, and SNAIL in RAW264.7 macrophages (Figure 1C). The nuclear to cytoplasmic ratio of LSD1 (Fn/c) was almost equal, suggesting downregulation of LSD1 in both the cytoplasmic and nuclear compartments on phenelzine treatment (Supplementary Figure 1). Conversely, treatment with either GSK or M2 polarization with IL-4 had the opposite effect, with enhanced cell number and expression intensity of CoREST and LSD1 and an overall increase in expression of CoREST, LSD1, and SNAIL in both the cytoplasmic and nuclear compartments. However, in this case, the Fn/c of LSD1 was clearly nuclear biased in the control group and increased further by M2 polarization or GSK treatment, perhaps by stabilizing LSD1 in the nucleus and enhancing expression (Figure 1C).

Phenelzine treatment abrogated co-localization of LSD1 and CoREST or SNAIL and CoREST as indicated by a strong negative PCC score in the phenelzine-treated samples (Figure 1D). GSK induced the opposite, with LSD1 and CoREST or SNAIL and CoREST strongly co-localizing with a positive PCC score (Figure 1D).

Overall, these data suggest that M1 (IFN-γ + LPS) polarization destabilizes and globally reduces LSD1, SNAIL, and CoREST expression, the overall cell population expressing these markers, and CoREST/LSD1 and CoREST/SNAIL complexes. Cells treated with phenelzine mimics this phenotype, impacting on both the FAD and CoREST domains of LSD1. Conversely, GSK or M2 (IL-4) polarization stabilizes and induces nuclear LSD1 and CoREST expression by enhancing their spatial co-localization. This suggests that overall that phenelzine inhibition aligns with M1 (IFN-γ + LPS)—macrophage polarization in the context of inhibiting both the catalytic FAD and nuclear CoREST domain of LSD1 Nuclear LSD1 activity can determine the macrophage phenotype.

**Figure 2:**
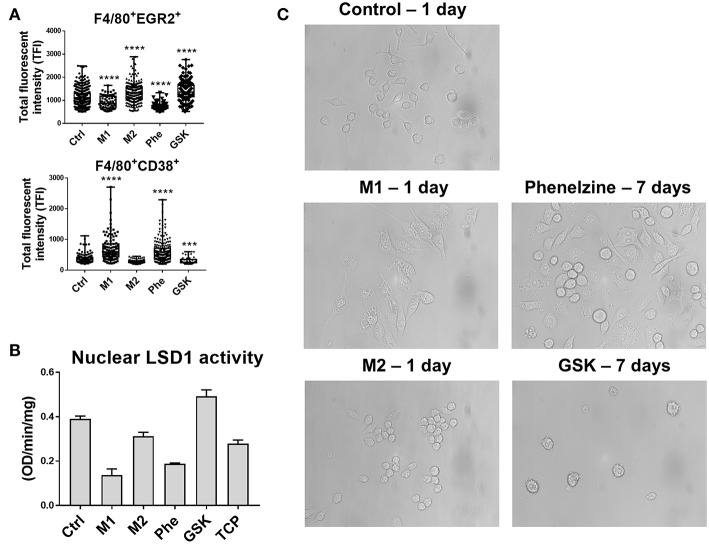
Phenelzine or M1 polarization upregulates M1 protein CD38 and targets the nuclear activity of LSD1. RAW264.7 cells were treated with LPS + IFN-γ, IL-4, or 500 μM phenelzine or GSK for 24 h. Protein targets **(A)** EGR2 and CD38 in F4/80^+^ cells were localized by confocal laser scanning microscopy. Single 0.5 μm optical sections were obtained using an Olympus-ASI automated microscope with 100x oil immersion lens running ASI software. The final image was obtained by employing a high-throughput automated stage with ASI spectral capture software. Digital images were analyzed using automated ASI software to determine the distribution and intensities automatically with automatic thresholding and background correction. Graphs represent either a dot plot of the individual cell intensities or the average TFI (*n* = 2,000 cells). **(B)** LSD1 activity assay on nuclear extracts of RAW264.7 cells either untreated, M1/M2 polarized, or treated with phenelzine, GSK, or LSD1 inhibitor tranylcypromine. **(C)** Images of RAW264.7 cells treated with vehicle control, LPS + IFN-γ (M1), and IL-4 (M2) for 24 h. Phenelzine and GSK treated cells did not show morphology changes in 24 h (data not shown). In comparison, cells were treated with Phenelzine or GSK for 7 days.

To address the impact of LSD1 on macrophage polarization, we employed high-throughput, ASI Digital Pathology Platform which allows both the quantification of immuno-fluorescent intensity and population distribution of stained cells using proprietary algorithms developed in partnership with ASI:Metagene using automatic autofluorescence correction with automatic signal intensity and cell detection to detect up to 6 colors plus DAPI. This system was employed to analyze both the expression and population distribution of the M1 marker CD38 and M2 marker EGR2 in RAW264.7 mouse macrophages treated with phenelzine, GSK, or cytokine-induced M1 (IFN-γ + LPS) or M2 (IL-4) phenotypes. Treatment with M1 (IFN-γ + LPS) or phenelzine reduced expression of EGR2 (an M2 marker) and the overall percentage of cells positive for EGR2 in F4/80^+^ RAW264.7 cells (Figure 2A), whereas induction with M2 (IL-4) or treatment with GSK induced expression of EGR2 and increased the percentage of EGR2^+^ cells (Figure 2A). Conversely, phenelzine treatment or M1 (IFN-γ + LPS) induction increased expression of the M1 marker CD38 and increased the proportion of CD38^+^ cells (Figure 2A), and M2 (IL-4) polarization and GSK treatment significantly reduced both expression of CD38 and the percentage of CD38^+^ cells. Thus, different LSD1 inhibitors have different and opposing effects on macrophage polarization.

We previously reported the importance of nuclear LSD1 phosphorylation at serine 111 (LSD1-s111p) in both CSCs and macrophages within the TME (22). We also showed that LSD1 inhibition significantly reduces LSD1-s111p and the transcription factor SNAIL expression in circulating tumor cells (CTCs) (22). We therefore sought to determine the impact of these LSD1 inhibitors on LSD1 activity and how M1 (IFN-γ + LPS) or M2 (IL-4) macrophage polarization affects the nuclear distribution of LSD1-s111p using high-resolution immunofluorescent microscopy for LSD1-s111p, histones modifications H3K9Me2 and H3K4me2, and SNAIL in RAW264.7 macrophage nuclei. H3K4 and H3K9 are direct LSD1 targets, and overexpression of the transcription factor SNAIL is associated with M2-like macrophage polarization (19, 42). Phenelzine treatment significantly reduced nuclear LSD1-s111p levels to like those seen in M1-polarized macrophages and enhanced levels of H3K9me2 and H3K4me2 (Supplementary Figure 1). Conversely, there was increased LSD1-s111p and decreased H3K9me2 and H3k4me2 levels in M2 and GSK-treated cells (Supplementary Figure 1). Phenelzine also reduced LSD1 nuclear enzymatic activity. Polarizing cells toward an M1 phenotype using IFN-γ and LPS also reduced nuclear LSD1 activity compared to controls (Figure 2B). However, treatment of cells with IL-4 (M2) and GSK did not inhibit the nuclear activity of LSD1 (Figure 2B). Interestingly, we also observed similar morphological changes between IFN-γ + LPS and phenelzine treatment after 7 days (phenelzine and media changed every 2 days) (Figure 2C).

Therefore, phenelzine can target nuclear LSD1 activity and have a role in initiating/priming macrophage polarization that is likely a pre-requisite for initiating phagocytosis.

### Phenelzine Treatment Can Reprogram Macrophages to Exhibit M1-Like Gene Signatures With PD1, PD-L1, and PD-L2 Checkpoint Expression

**Figure 3:**
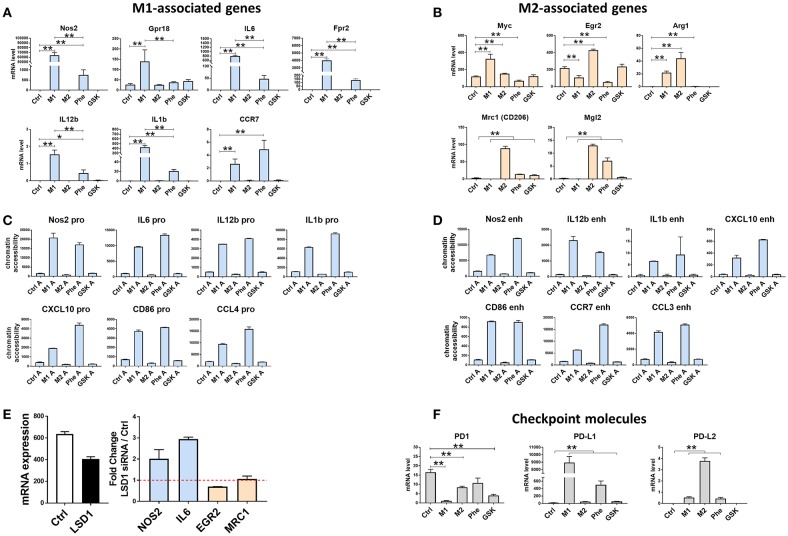
LSD1 can regulate genes associated with macrophage polarization toward an M1 phenotype and checkpoint molecules. RAW264.7 cells were untreated or treated with LPS + IFN-γ (M1), IL-4 (M2), or 500 μM of phenelzine or GSK for 24 h. Quantitative real-time PCR of genes associated with **(A)** M1-associated genes and **(B)** M2-associated genes were used to compare different treatment groups. Graphs are represented as mRNA levels normalized to GAPDH. Graphs show means ± SE (n = 3). ^*^p < 0.05, ^**^p < 0.005 Mann-Whitney t-test. **(C)** and **(D)** shows chromatin accessibility of genes in the promoter **(C)** and enchancer **(D)** regions of associated genes using quantitative real-time PCR using FAIRE samples. **(E)** 10nM LSD1 siRNA transfected cells and **(F)** checkpoint molecules and were used to compare different treatment groups.

Given the similarities between M1 (IFN-γ + LPS)-polarized macrophages and macrophages treated with phenelzine, we next determined whether LSD1 inhibition with phenelzine and GSK mimic polarized macrophage gene signatures. The gene expression of phenelzine-treated cells was similar to the M1 phenotype (*Nos2, Gpr18, IL6, Fpr2, IL12b, ILl1b*, and *Ccr7*) (12) induced by IFN-γ and LPS (Figure 3A). This corresponded to increased accessibility at the promoter (Figure 3C) and enhancer (Figure 3D) regions of those M1-like genes in the M1 (IFN-γ + LPS) or phenelzine treated RAW264.7 cells. In addition, genes associated with the M2 phenotype (*Myc, Egr2, Arg1, Mrc1*, and *Mgl2*) were expressed at much lower levels compared to cells polarized toward an M2 phenotype using IL-4 (Figure 3B). GSK-induced gene signatures, on the other hand, did not show a similar correlation with M1-polarized cells (Figures 3A,B).

In order to confirm these effects were due to LSD1 inhibition, we knocked-down LSD1 in RAW264.7 cells with siRNAs. This resulted in a 40% inhibition of LSD1 gene expression (Figure 3E) and an increase in key M1 markers such as *Nos2* and *Il-6*, a decrease in M2 markers *Egr2* and no change in *Mrc1* (CD206) (Figure 3E).

Therefore, inhibiting the catalytic FAD and nuclear CoREST domain of LSD1 with phenelzine can upregulate M1-associated genes and decrease M2-associated genes, while inhibition of the FAD domain (GSK) alone does not. This indicates an important role for both the FAD domain of LSD1 and its stabilization by CoREST in regulating genes associated with M1 macrophages.

Targeting the PD1-PD-L1 axis is an effective therapeutic approach in cancer, and macrophages express these checkpoint molecules (43, 44). Unpolarized and M1 (IFN-γ + LPS)- and M2 (IL-4)-polarized RAW264.7 cells express different levels of PD1, PD-L1, and PD-L2, so given the effect of LSD1 inhibition on macrophage polarization, we also wanted to determine the effect of LSD1 inhibition on the PD1-PDL1/2 axis.

*Pd1* expression was generally lower in all treated cells compared to controls (Figure 3F), with M1 (IFN-γ + LPS)-polarized cells expressing the lowest *Pd1* (Figure 3F), Interestingly, we observe high enrichment of *Pd-l1* (approximately 450-fold) and *Pd-l2* (approximately 3-fold) in M1 (IFN-γ + LPS) and M2 (IL-4)-polarized cells, respectively compared to control (Figure 3F). Phenelzine-treated macrophages displayed similar *Pd-l1* and *Pd-l2* expression to M1 (IFN-γ + LPS)-polarized cells except for *Pd1* (Figure 3F). GSK treatment, however, mimicked an M2 (IL-4)-type expression pattern of *Pd-l1* but not *Pd1* and *Pd-l2* (Figure 3F).

These data suggest that macrophage polarization may contribute to *Pd-l1* and *Pd-l2* expression at both the gene and protein level, with M1 (IFN-γ + LPS)-polarized cells expressing higher *Pd-l1* levels and M2 (IL-4)-polarized cells expressing higher *Pd-l2* levels. Phenelzine treatment appears to mimic this M1(IFN-γ + LPS)-like checkpoint protein expression but GSK induces greater variability, suggesting that other post-translational mechanisms may be involved.

### Phenelzine Treatment Can Produce More Favorable Macrophage Signatures in the TME That Mimic Those Seen With Protein-Bound Paclitaxel (Abraxane) and PD1-Based Immunotherapy

**Figure 4:**
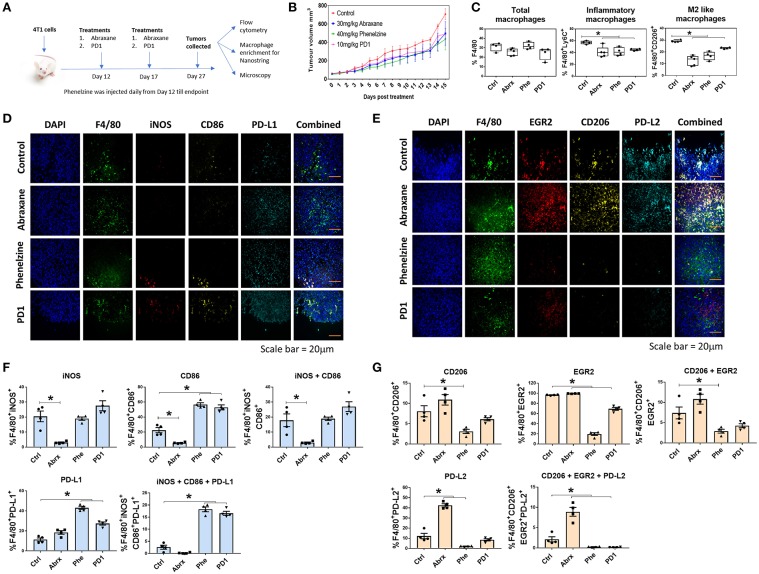
Phenelzine treatment polarizes macrophages in the tumor microenvironment toward an M1 phenotype. **(A)** Treatment regime using the BALB/c 4T1 breast cancer model. **(B)** Tumor volumes of mice treated with vehicle control, Abraxane, Phenelzine or PD1 (*n* = 4/5). **(C)** Flow cytometry for total macrophages, inflammatory macrophages, and M2-like macrophages in the TME. ^*^p < 0.05, Mann–Whitney *t*-test (*n* = 4/5). Representative images of **(D)** M1 and **(E)** M2 staining of FFPE tumor tissues in 4T1 mouse model. **(F)** Sections of primary 4T1 tumors were fixed and IF microscopy performed probing with M1 focused primary antibodies to F4/80, iNOS, CD86, and PDL1 with DAPI (green = F4/80 red = iNOS, yellow = CD86, cyan = PDL1, blue = DAPI). The population % of F4/80 cells positive for iNOS, CD86 and PDL1 was measured using ASI's mIF system. Representative images for each dataset are shown. Graphs plots represent the % population (*n* ≥ 500 cells profiled per a group, *n* = 4 mice). **(G)** Section of primary 4T1 tumors were fixed and IF microscopy performed probing with M2 focused primary antibodies to F4/80, EGR2, CD206, and PDL2 with DAPI (green = F4/80 red = EGR2, yellow = CD206, cyan = PDL2, blue = DAPI). The population % of F4/80 cells positive for EGR2, CD206, and PDL2 was measured using ASI's mIF system. Representative images for each dataset are shown. Graphs plots represent the % population (*n* ≥ 500 cells profiled per a group, *n* = 4 mice).

We next sought to determine if phenelzine treatment also reprograms macrophages in the TME of cancers in mice. Since chemotherapy is the standard of care for breast cancer patients and given our results on PD1 expression in response to phenelzine treatment, we also treated syngeneic TNBC 4T1 mice with protein-bound paclitaxel (Abraxane) and PD1 immunotherapy (Figure 4A). Phenelzine, Abraxane and PD1 reduced tumor volumes (Figure 4B) compared to controls, however, this difference was not significant.

There were no significant differences in total F4/80^+^ macrophages between treatment groups (Figure 4C). However, all three treatments induced significantly lower proportions of inflammatory (F4/80^+^Ly6C^+^) and M2-like macrophages (F4/80^+^CD206^+^) (Figure 4C). We next quantified F4/80 and M1-like markers (iNOS, CD86 and PD-L1) (Figure 4D) or M2-like markers (EGR2, CD206, and PD-L2) (Figure 4E) in tissue sections from individual tumors using the high-throughput, ASI Digital Pathology Platform as described above in section Phenelzine Treatment Can Reprogram Macrophages to Exhibit M1-Like Gene Signatures With PD1, PD-L1, and PD-L2 Checkpoint Expression (Figures 4F,G). There was a significant increase of F4/80 macrophages expressing three M1-like markers with phenelzine and PD1 treatment alone compared to control and Abraxane treated mice (Figure 4F). Further, there was a significant decrease in F4/80 macrophages expressing all three M2-like markers with phenelzine and PD1 treatment compared to control and Abraxane treated mice (Figure 4G). Interestingly, treatment with Abraxane alone decreased the number of macrophages expressing the M1-like markers and significantly increased the number of macrophages expressing M2-like markers (Figures 4F,G).

**Figure 5:**
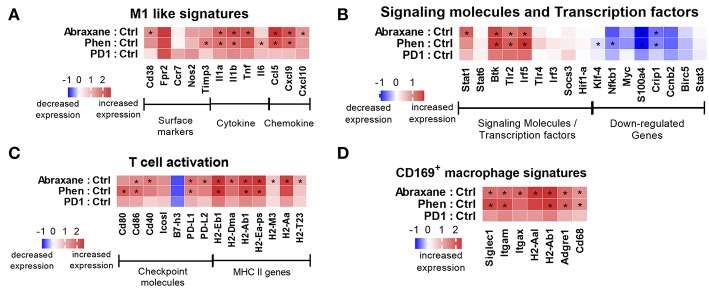
Nanostring counts (log2 fold-change) from RNA isolated from macrophages in the TME for **(A)** M1 phenotypic signatures and pathways, **(B)** M1 signaling molecules and transcription factors, **(C)** T cell activation gene signatures, and **(D)** CD169^+^ macrophage gene signatures. ^*^Indicates Benjamini–Yekutieli false discovery rate value < 0.05.

The macrophages were then analyzed for innate immunity pathways (770 genes, 19 default pathway annotations) using the NanoString platform. Phenelzine, Abraxane, and PD1 all modulated key M1 gene signatures compared to macrophages from control mice (Figures 5A,B), although PD1-related changes were non-significant.

Macrophages are professional antigen-presenting cells and express various co-stimulatory molecules that help with antigen presentation to T cells via MHC class II (45, 46). Phenelzine or Abraxane upregulated some MHC II genes, positive co-stimulatory genes such as *Cd80, Cd86, Cd40*, and *Icos-l*, and downregulated negative regulators such as *B7-H3* in macrophages in the TME (Figure 5C). Genes were also upregulated in a subset of CD169^+^ macrophages (Figure 5D). Therefore, phenelzine had a significant impact on the genetic reprogramming of macrophages toward a more M1-like phenotype in the TME in mice.

### The Macrophage Post-translational Modification Landscape of Genes Up-Regulated in Phenelzine

We next determined how phenelzine treatment affected macrophage gene expressions measured using the NanoString platform (FDR < 0.15) by overlaying these data with published epigenomic data.

**Figure 6:**
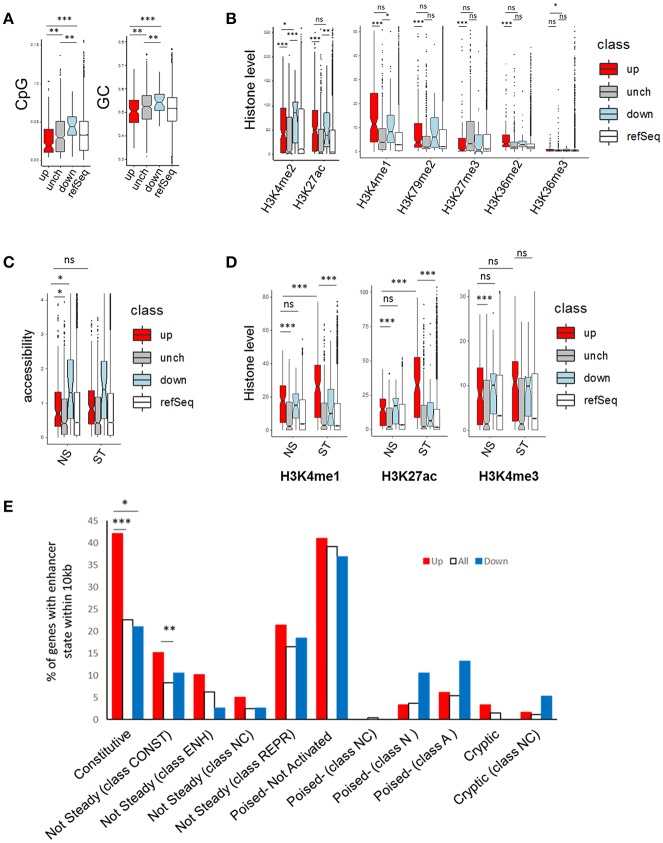
CpG content, GC content, histone marks, and accessibility of genes in the NanoString panel. **(A)** CpG and GC content, **(B,D)** histone levels and **(C)** accessibility of genes ± 1 kb from transcription start site (TSS) of NanoString genes up-regulated, downregulated (FDR < 0.15), or unchanged (FDR > 0.15) and genes from refSeq in RAW264.7 cells (47). **(C)** shows accessibility of Nanostring genes against gene sets from bone marrow derived macrophages (BMDMs) with or without 6 h of LPS stimulation (48). **(E)** Levels of up/down regulated NanoString genes that had enhancers within 10 kb of their TSS from BMDMs with or without 24 h of LPS stimulation (31). A *t*-test with unequal variance (Welch two sample *t*-test) was used with ^*^p < 0.05, ^**^p < 0.01 and ^***^p < 0.001.

The 178 genes up-regulated by phenelzine had promoters (±1 kb TSS) with significantly (*p* < 0.01) less CpG and GC content than either the “unchanged” or down-regulated genes (Figure 6A). The 38 down-regulated gene promoters had significantly (*p* < 0.01) more CpG and GC content than unchanged gene promoters (Figure 6A).

Up-regulated gene promoters had significantly less H3K4me3 (*p* < 0.05) but not H3K27ac and H3K4me1 than the down-regulated gene promoters, in resting RAW264.7 cells (Figure 6B) (47). Importantly the up-regulated gene promoters had less H3K27me3 than the unchanged genes, suggesting their non-maximal gene expression is not due to H3K27me3-mediated repression (Figure 6B).

Further, the up-regulated promoters were less accessible in both NS and 6 h LPS-stimulated bone marrow derived macrophages (BMDMs) than the down-regulated genes but were more accessible than the unchanged genes (Figure 6C) (48).

The up-regulated promoters had significantly higher H3K4me1, H3K27ac, and H3K4me3 levels than the unchanged genes in BMDMs (31) and H3K4me1 and H3K27ac levels significantly increased (1.6-fold and 2.3-fold respectively) after 24 h LPS stimulation (Figure 6D).

Using enhancer regions identified in Ostuni et al. (31), we determined how many of the up- and down- regulated genes had enhancers within 10 kb of their TSS. Significantly more of the up-regulated genes had constitutive or constitutive but not steady (24 h_CONST) macrophage enhancers than all the entire set of genes. This is consistent with our chromatin accessibility profiles in Figures 3C,D.

### LSD1 Inhibition and Chemotherapy Target the Hippo and Wnt Signaling Pathways

**Figure 7:**
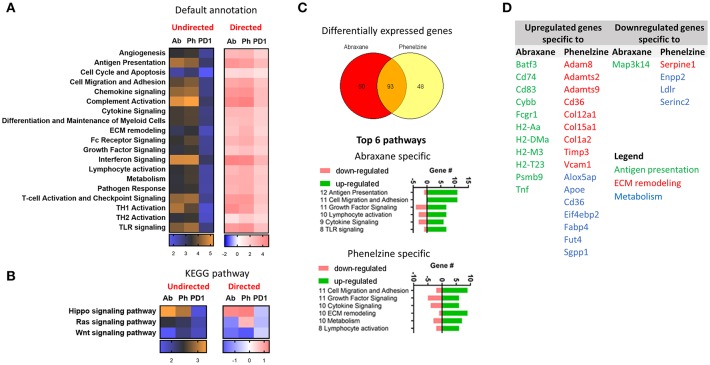
Phenelzine affects Hippo, Wnt, and Ras signaling pathways and genes associated with ECM remodeling and metabolism. **(A)** Heatmap displaying the undirected and directed global significance score statistics using the default and **(B)** KEGG pathway annotations. Undirected scores measure the extent of differential expression of a geneset's genes against control ignoring whether each gene within the set is up- or downregulated. Orange denotes genesets whose genes exhibit extensive differential expression against control, and blue denotes genesets with less differential expression. Directed scores measure the extent to which a geneset is up- or downregulated compared to control. Red denotes genesets that show extensive overexpression and blue denotes genesets with extensive underexpression. **(C)** Venn diagram indicating genes that are differentially expressed with a false discovery rate of < 0.05 (Benjamini-Yekutieli) and top 6 pathways that those genes are fall under. **(D)** Table showing the upregulated and downregulated genes that are specific to Abraxane and phenelzine.

Using the NanoString platform, there was higher differential expression (DE) of NanoString default geneset annotations between mice treated with Abraxane and phenelzine than to control (orange) than with PD1 (blue) (Figure 7A, undirected). The complement activation, interferon and chemokine signaling, T-cell activation and checkpoint signaling, Th1 activation, antigen presentation, and TLR signaling pathways had higher DE (orange) (Figure 7A). Phenelzine showed similar upregulation of pathways as macrophages from Abraxane-treated mice (Figure 7A, directed). When the gene signatures were annotated using KEGG, Abraxane-, and phenelzine-treated groups upregulated genes associated with the Hippo signaling pathway and downregulated genes associated with the Wnt signaling pathway (Figure 7B). Phenelzine treatment also upregulated genes linked to the Ras signaling pathway, distinct from Abraxane- and PD1-treated mice (Figure 7B).

We next determined which genes were specific to Abraxane and phenelzine treatments. The treatments shared 93 gene signatures, 50 specific to Abraxane and 48 specific to phenelzine (Figure 7C). The top six NanoString default pathways specific to Abraxane or phenelzine are shown in Figure 7C. Abraxane treatment seemed to have a greater impact on genes associated with antigen presentation that phenelzine (Figures 7C,D), while phenelzine had a greater effect on genes associated with extra-cellular matrix remodeling and metabolism (Figures 7C,D).

Although macrophages isolated from the TME of mice treated with Abraxane and phenelzine showed similar gene expression changes such as upregulation of M1-like genes and pathways, these two different treatments also target specific pathways: antigen presentation in the case of Abraxane-treated mice and ECM remodeling and metabolism in phenelzine-treated mice.

## Discussion

Macrophages form a large component of the TME and may have anti- or pro-tumorigenic properties, making them a viable target for cancer immunotherapy. Macrophages are broadly described as M1 (classical) or M2 (alternative) depending on their activation, although this is known to represent a phenotypic spectrum. Here we examined the effects of epigenetic inhibition of LSD1 on macrophage phenotype *in vitro* and *in vivo* using two different LSD1 inhibitors: GSK, which only binds to the FAD domain, and phenelzine, which can bind to the FAD domain and disrupt the LSD1-CoREST complex. Using these inhibitors, we show for the first time a potential role for the FAD and LSD1-CoREST complex in mediating downstream gene signatures to generate an M1-like macrophage phenotype *in vitro* and in the TME of mouse triple-negative breast cancers.

To understand how LSD1 inhibition could affect macrophage polarization, we utilized two LSD1 inhibitors, GSK and phenelzine. While both inhibitors bound to the FAD domain, phenelzine but not GSK induced small structural changes in the CoREST binding region of LSD1, which has been shown to be important for LSD1's activity and stability (49). Phenelzine disrupted co-expression of nuclear LSD1 and CoREST and SNAIL and CoREST. Interestingly, when cells were polarized toward an M1 phenotype using IFN-γ and LPS, there was similar downregulation of nuclear LSD1 and CoREST, but this did not occur in M2 polarization with IL-4 or with GSK treatment. Therefore, phenelzine may play a dual role by disrupting the LSD1-CoREST complex and potentially its stability and activity, while GSK can only bind the FAD domain. The LSD1-CoREST complex also appears to have a role in repressing the M1 macrophage phenotype, because there was similar downregulation of LSD1, CoREST, and SNAIL expression when the cells were polarized toward an M1 phenotype using IFN-γ and LPS. However, this was not observed when cells were polarized to an M2 phenotype or treated with GSK, which only targets the FAD domain. This highlights the importance of targeting both the FAD and CoREST domains of LSD1 to reprogram macrophages toward an M1-like phenotype for therapeutic benefit.

The LSD1-CoREST complex has been shown to promote demethylation of nucleosomal histone 3 lysine 4 (H3K4) (49, 50) and histone 3 lysine 9 (H3K9) (51). To determine if LSD1 demethylase activity participates in macrophage polarization, we used immunofluorescence microscopy to show that cells treated with phenelzine had higher expression of histone H3K9me2 and H3K4me2, which are direct targets of LSD1. Macrophages polarized to an M1 phenotype using IFN-γ and LPS showed similar higher expression of these histone markers, with the opposite true in cells polarized to an M2 phenotype or treated with GSK. We also measured nuclear LSD1 demethylase activity in our *in vitro* model and showed that phenelzine, tranylcypromine (an LSD1 inhibitor), and macrophages polarized with IFN-γ and LPS inhibited LSD1 activity compared to control cells, whereas treatment with GSK or M2 polarization had little effect on nuclear LSD1 activity. This could potentially be due to several factors; for example, LSD1-CoREST complex disruption by phenelzine or IFN-γ and LPS, could destabilize the LSD1 protein in addition to inhibiting the FAD enzymatic domain. Signaling through the IFN-γ receptor by IFN-γ and toll-like receptors (TLR) by LPS could also impede LSD1 demethylase activity on H3K4me2 and H3K9me2. Interestingly, we also observed that RAW264.7 cells had similar cell morphology after 7 days of treatment to cells treated with IFN-γ and LPS (M1). This did not occur after 24 h of treatment (data not shown), suggesting that phenelzine might prime the macrophages to differentiate into a similar morphology to M1 treated cells. As predicted, the expression of nuclear phosphorylated LSD1 at serine 111 (LSD1-s111p) is lower in macrophages polarized to M1 (IFN-γ + LPS) or treated with phenelzine. This most likely the result of the loss of LSD1 due to the destabilization of the LSD1-CoREST complex.

We also showed that M1 (IFN-γ + LPS)-polarized and phenelzine-treated macrophages downregulate expression of the transcription factor SNAIL, and previous work has shown that SNAIL knockdown in human THP-1 macrophages and breast cancer cells promotes M1 polarization both *in vitro* and *in vivo* (42, 52). Therefore, inhibition of the demethylase activity of LSD1 using phenelzine, which targets both the FAD and CoREST domains, could play a role in M1 polarization, either directly or indirectly through the transcription factor SNAIL. Intriguingly, we have previously shown that this nuclear phosphorylated form of LSD1-s111p is mediated by protein kinase-C theta (PKC-θ) in cancer stem cells (CSCs) (22, 53). PKC-θ has been reported to regulate various genes in T cells (54) and promotes a potent pro-inflammatory macrophage phenotype (55). However, this latter study may not have examined the nuclear role of PKC-θ, so it could be possible that in the context of LSD1-s111p, the nuclear role of PKC-θ is distinct from its cytoplasmic role as previously shown in CSCs (22). We have also previously shown that nuclear PKC-θ can regulate microRNAs in T cells (56). Therefore, it would be interesting to explore the nuclear role of PKC-θ and its ability to mediate the M1 phenotype via LSD1.

The classically activated M1 (IFN-γ + LPS) phenotype has been shown to have anti-tumorigenic properties. We found that the gene signatures of RAW264.7 mouse macrophages inhibited with phenelzine mimicked the M1-like signatures of macrophages classically activated with IFN-γ and LPS. It has previously been shown that the increase in demethylase Jumonji domain containing 3 (Jmjd3) contributes to the decrease in H3K27me2/3 and transcriptional activation of specific M2 marker genes such as *Chi3l3, Retnla*, and *Arg1* (57). Our results show that LSD1, another demethylase, might play a role in regulating macrophage polarization toward an M1 phenotype. Of therapeutic relevance, we also discovered that PD1, PD-L1, and PD-L2 might also change when macrophages polarize, with unpolarized cells expressing PD1, M1 cells expressing PD-L1, and M2-polarized cells expressing PD-L2. Therefore, these immune checkpoint proteins might be useful M1 or M2 biomarkers.

We hypothesized that treatment of tumor-bearing mice with phenelzine could alter the TAMs in the TME. Using a TNBC syngeneic mouse model, we showed that LSD1 inhibition slightly reduced tumor volume and epigenetically reprogrammed TAMs to a more anti-tumor phenotype. While there was no change in the total F4/80^+^-expressing macrophage population, there was a significant reduction in both inflammatory (Ly6C^+^) and M2-like macrophages (CD206^+^). We postulated that since there were no significant changes in the total macrophage population, phenelzine treatment reprogrammed the macrophages already present in the tumor toward an anti-tumor phenotype. Interestingly, this effect was also seen with Abraxane and anti-PD1 antibody, suggesting that phenelzine alone was able to contribute to this reprogramming at the gene level. Our tissue section of mice tumors showed that phenelzine and PD1 treated mice tumors contained more macrophages expressing M1-like markers (iNOS, CD86, and PD-L1) and lower proportions of M2-like markers (CD206, EGR2, and PD-L2) suggesting that phenelzine and PD1 treatment favors a M1-like phenotype in the TME.

To further characterize the TAMs in phenelzine-treated mice, we used the NanoString platform to show that they altered expression of genes related to an M1 phenotype such as *Il1a, Il1b, Il6, Ccl5, Cxcl9*, and *Cxcl10* (58–62). There was also increased expression of *Stat1* and decreased expression of *Stat3*, which are associated with M1 and M2 polarization, respectively (58, 63, 64). Macrophages from phenelzine-treated mice also showed a significant decrease in the NFκB1 transcription factor compared to control. It has been shown that blocking NFκB signaling can switch TAMs to an M1-like phenotype (65) and that p50 overexpression in TAMs inhibits M1 anti-tumor resistance (66). It is known that the NFκB signaling pathway activation through TLRs induces M1 macrophage polarization and subsequent pro-inflammatory effects through the p65 phosphorylation and IκB (67–69), so it would be interesting to determine whether the TLR4/NFκB signaling is affected by phenelzine treatment. Phenelzine treatment also significantly reduced KLF4 expression in macrophages isolated from the TME, with KLF4 previously shown to be reduced in M1 macrophages and robustly induced in M2 macrophages (70) via the RORα (71) and IRF4 axes (72). We also saw a significant increase in IRF5 expression, another M1-associated protein, on phenelzine treatment (63, 73, 74) and Btk was similarly increased; Btk inhibition with ibrutinib impairs M1 polarization (75, 76). Phenelzine-treated macrophages also significantly increased TIMP3, which is a potent tumor angiogenesis and growth inhibitor (77–79). We have previously shown using LSD1 chromatin immunoprecipitation (ChIP) sequencing that LSD1 can directly or indirectly execute genome-wide EMT via target transcription factors (22). Therefore, it is interesting to observe common mechanisms affecting gene regulation in CSCs and M1 polarization.

Of note, phenelzine-treated macrophages had similar features to the CD169+ macrophages that dominate anti-tumor immunity via cross-presentation to cytotoxic T lymphocytes (80–84). LSD1 inhibition also upregulated checkpoint molecules such as CD80/86 and MHC class II genes and downregulated negative regulators such as B7-H3. Consistent with our *in vitro* polarization studies, phenelzine significantly upregulated PD-L1. Although PD-L1 is usually an inhibitory signal, it was upregulated when macrophages were polarized toward an M1 phenotype, and a similar trend was also seen in macrophages isolated from Abraxane- and PD1-treated mice.

Our *in silico* analysis also showed that genes upregulated by phenelzine treatment had promoters with significantly less CpG and GC content compared to “unchanged” or downregulated genes. Conversely, downregulated gene promoters had significantly more CpG and GC content. A direct repressive role for LSD1 for the M1 genes is more likely to be due to its demethylation of H3K4. High H3K4 methylation is associated with increased DNA accessibility at promoters and enhancer regions] and the phenelzine responsive gene promoters are initially less accessible and more tilted toward a lower H3K4 methylation state than the down-regulated genes. In LPS activated cells the H3K4 methylation levels at the phenelzine responsive gene promoters increases. We also show that phenelzine treatments increases accessibility at both the promoters and nearby enhancers which is mostly likely linked to increased methylation of the surrounding histones. H3K4 methylation is dependent, not only on demethylases but also on methylases like MLL1 and SET1 (85). MLL1 contains a CpG binding domain, and SET1 binds an accessory protein with one (85). It is possible that the H3K4 methylation levels of CpG low promoters are more dependent on the levels and activity of demethylases, while CpG high promoters are more dependent on levels and activities of methylases.

When examining pathway changes, phenelzine inhibition increased genes associated with the Hippo and Ras pathways but decreased genes associated with the Wnt pathway. Upregulated Hippo signaling sequesters β-catenin in the cytoplasm via YAP/TAZ, negatively regulating the Wnt pathway (86). Wnt/β-catenin signaling is activated via c-myc during monocyte to macrophage differentiation and M2 polarization (87). Active Wnt signaling is also implicated in macrophage-associated angiogenesis and tumor invasion (88–90). Therefore, LSD1 can play a role in regulating genes associated with both pathways, and LSD1 inhibition by phenelzine may be able to reduce M2 macrophage polarization as well as macrophage-associated angiogenesis and tumor invasion. Interestingly, we also observed the upregulation of genes associated with Ras signaling, a commonly dysregulated pathway in various cancer types that regulates cell growth, survival, proliferation, and apoptosis (91–93). How LSD1/CoREST destabilization in M1 macrophages upregulates Ras signaling would be worthy of further study.

Overall, Abraxane treatment affected genes associated with antigen presentation, whereas phenelzine affected genes associated with extracellular matrix (ECM) remodeling and metabolism. Therefore, LSD1 can modulate genes associated with ECM remodeling and metabolism, both important components of the TME (94–97). Since, ECM remodeling is mainly associated with M2 macrophages, it is plausible that phenelzine also impacts genes associated with M2 macrophages. Further studies should investigate whether this has a positive or negative functional impact in the context of TNBC. Macrophage function and polarization are also closely associated with metabolic functions, with the M1 inflammatory phenotype heavily dependent on glycolysis and M2 alternatively activated macrophages relying on oxidative phosphorylation [extensively reviewed in (98, 99)]. Since phenelzine treatment affects genes associated with metabolism, there may be the potential to epigenetically prime macrophages by modulating LSD1.

We have previously shown that LSD1 could target gene induction programs promoting epithelial to mesenchymal transition (EMT) and cancer stem cells (CSC) and that inhibition of LSD1 suppresses chemotherapy-induced EMT and cancer associated fibroblasts (CAFs) (22). However, it is also important to note that phenelzine could potentially affect other tumor infiltrating subsets of cells in the TME such as effector T-cells and myeloid-derived suppressor cells. This is beyond the scope of this study; however, it would be a very interesting area worth exploring.

**Figure 8:**
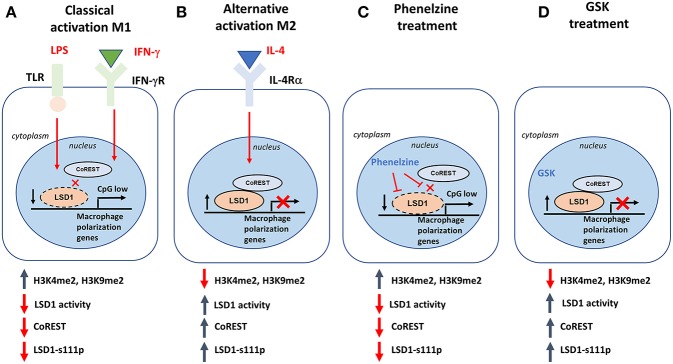
Putative model of how LSD1 can reprogram macrophage polarization. **(A)** When macrophages are stimulated with LPS and IFN-γ (classical activation; M1), disruption of CoREST destabilizes LSD1, which leads to LSD1 losing its repressive role in regulating M1-associated genes. It also increases LSD1 demethylase activity. **(B)** When macrophages are stimulated with IL-4 (alternative activation; M2), CoREST is not affected, resulting in stable expression of LSD1p and LSD1 maintaining its repressive role in regulating M1-associated genes. It also decreases LSD1 demethylase activity. **(C)** LSD1 inhibition using phenelzine can target both LSD1p and CoREST, mimicking a similar response to M1 polarization while **(D)** GSK was not able to achieve the same result because it did not disrupt the LSD1/CoREST complex.

Overall, our data proposes a model in which LSD1 poises M1-selective gene signatures in naïve macrophages by tethering to the epigenome of such genes, similar to in CSCs (22). LSD1 globally decorates the epigenetic landscape of M1 gene signatures in naïve macrophages with H3K4 and H3K9 methylation marks. Following activation of M1 signaling pathways, nuclear LSD1 activity is rapidly reduced due to the disassembly of the LSD1-CoREST complex, leading to destabilization of the nuclear LSD1 pool (Figure 8). This primes the epigenome of M1-inducible genes, leading to their expression. In parallel, M2 gene activation increases nuclear LSD1 activity and LSD1-CoREST, in turn maintaining repression of M1 genes and skewing induction of the M2 gene signature (Figure 8). Sequential ChIP and co-immunoprecipitation studies will be required to unravel the in-depth molecular signatures underlying LSD1's contribution to the M1/M2 phenotypes. Priming by phenelzine alone may not be sufficient to polarize macrophages toward a M1 phenotype, and further studies are needed to establish which combinatorial therapies optimally enhance the phenotypes observed in this study.

In conclusion, the LSD1 inhibitors phenelzine and GSK are useful tools for studying the catalytic and non-catalytic role of LSD1. These inhibitors have allowed us to dissect the contribution of LSD1 enzymatic activity and the nuclear LSD1-CoREST complex on M1/M2 phenotype switching. These effects were replicated *in vitro* and *in vivo*. Inhibitors with dual FAD and CoREST-targeting abilities could be important for reprogramming macrophages and potentially initiate an anti-tumor M1-like phenotype in TNBC and other cancers.

## Ethics Statement

This study was carried out in accordance with the recommendations of the Australian Code of Practice for the Care and Use of Animals for Scientific Purposes, Australian National University Animal Experimentation Ethics Committee. The protocol was approved by the Australian National University Animal Experimentation Ethics Committee.

## Author Contributions

AT, WT, RM, JF, and SR designed the experiments. AT, WT, RM, TD, ST, KH, and JK performed the experiments and analyzed the data. AT and SR wrote the manuscript. SR conceived the study.

### Conflict of Interest Statement

In accordance with NHMRC guidelines and our ethical obligations as researchers, we report that the University of Canberra, SR, RM, and AT have a financial interest in EpiAxis Therapeutics Pty Ltd. SR is also Chief Scientific Officer of EpiAxis Therapeutics Pty Ltd. We have in place a plan for managing any potential conflicts arising from that involvement. The remaining authors declare that the research was conducted in the absence of any commercial or financial relationships that could be construed as a potential conflict of interest.
