# Supplementary figures and images for: Lysine-Specific Histone Demethylase 1A Regulates Macrophage Polarization and Checkpoint Molecules in the Tumor Microenvironment of Triple-Negative Breast Cancer
Source: Front Immunol. 2019 Jun 12;10:1351. doi: 10.3389/fimmu.2019.01351 (PMC6582666; doi:10.3389/fimmu.2019.01351)

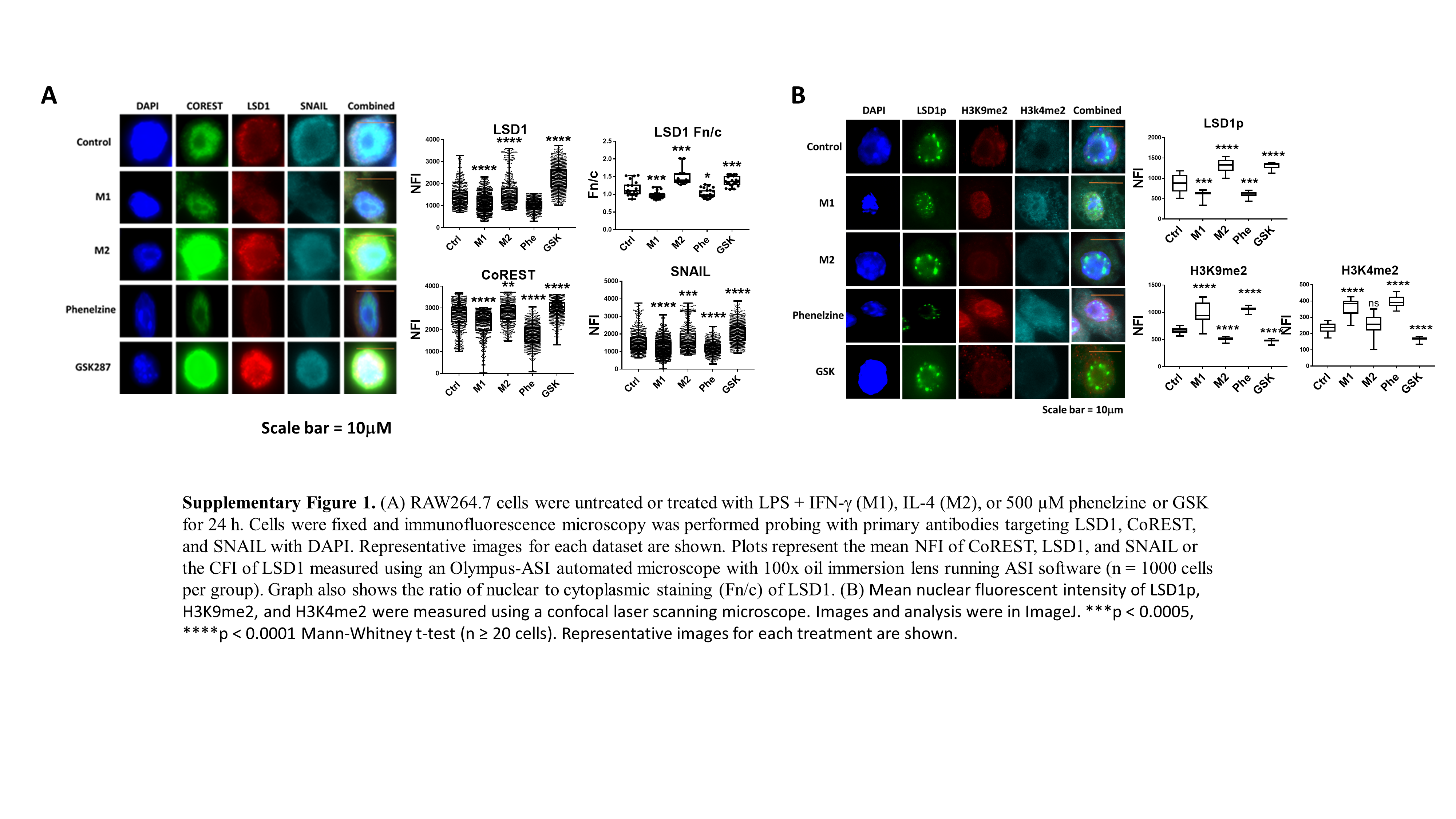

Supplement: Supplementary file 1 [file Image_1.TIF]

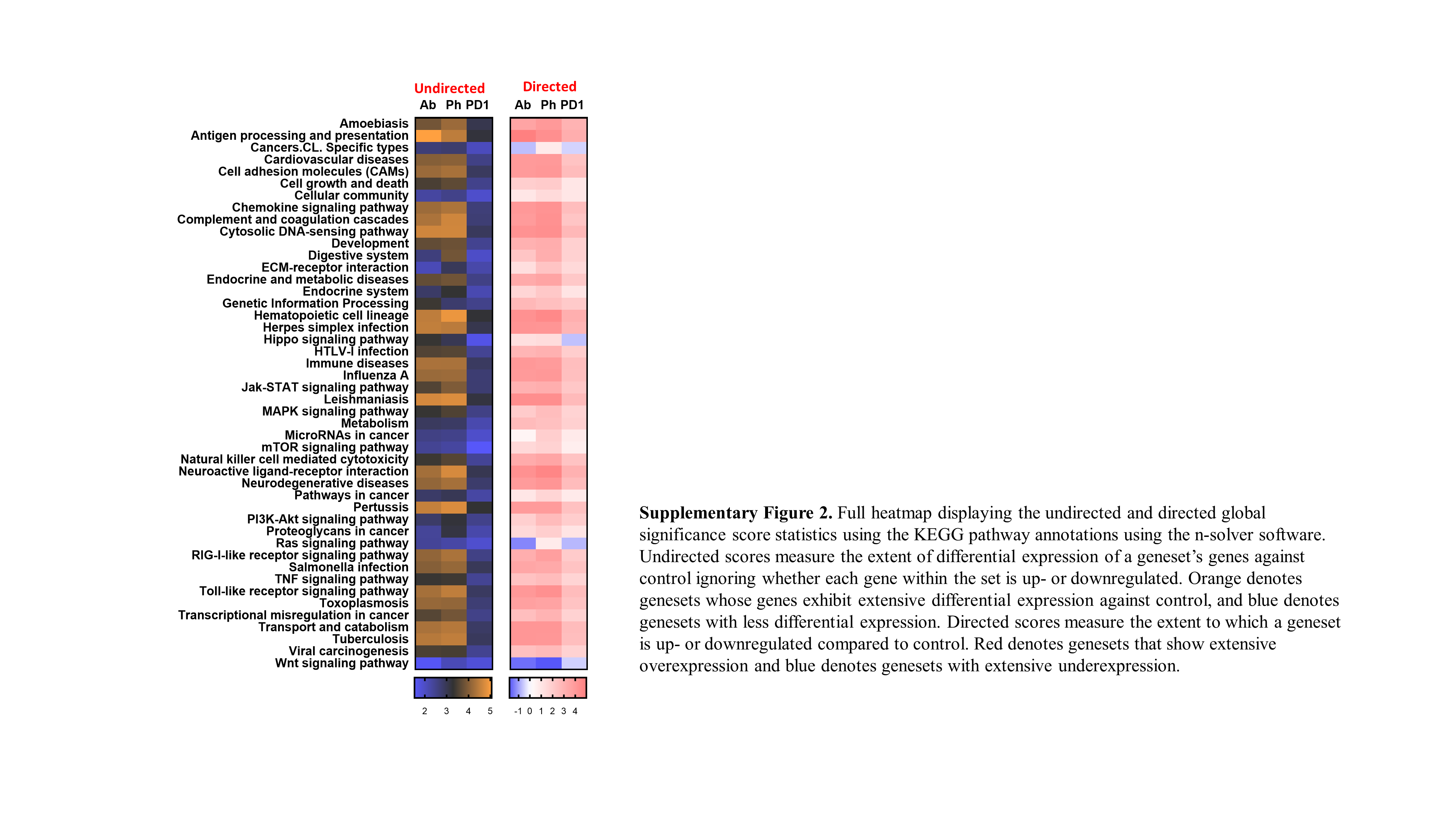

Supplement: Supplementary file 2 [file Image_2.TIF]

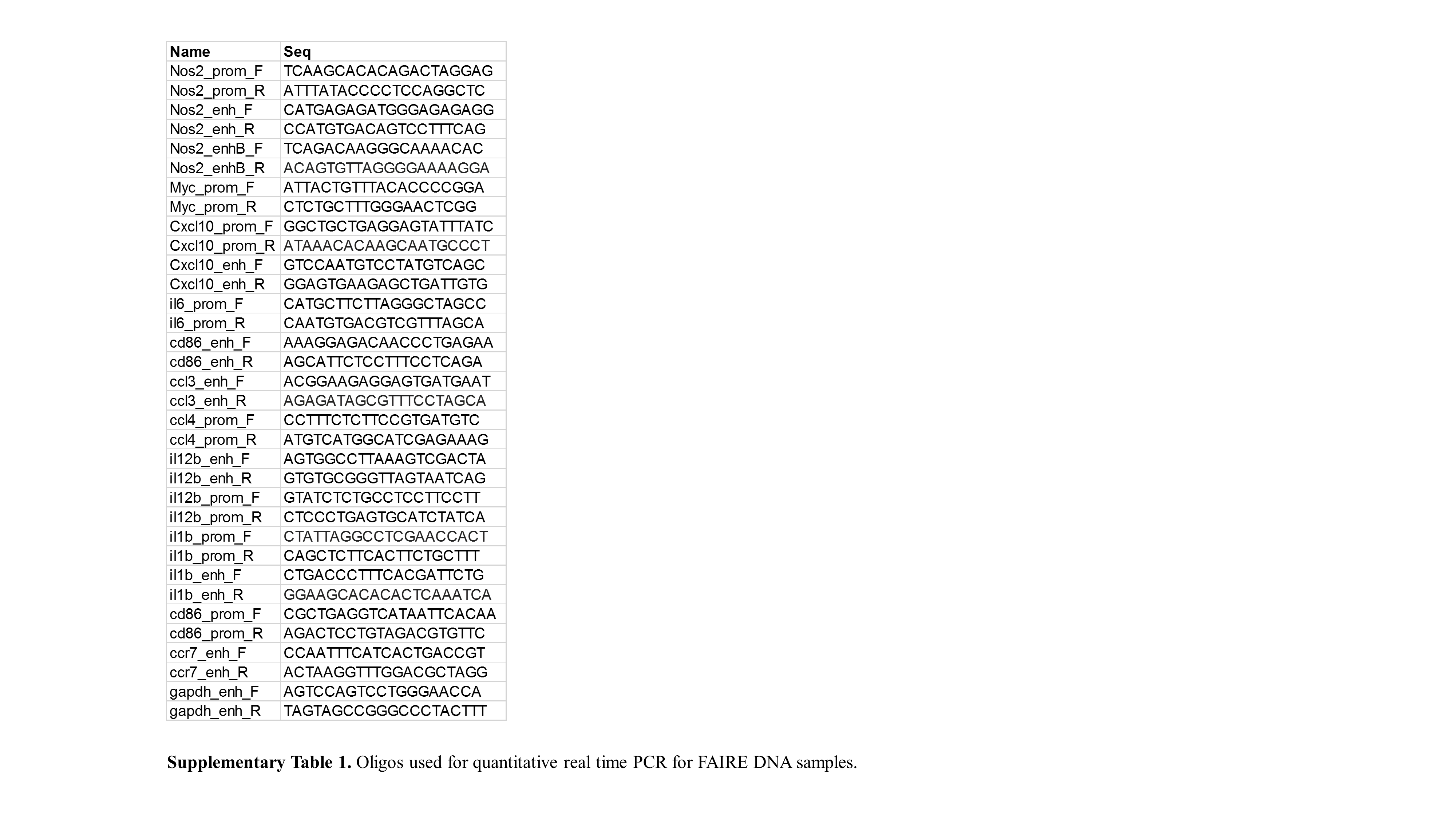

Supplement: Supplementary file 3 [file Image_3.TIF]

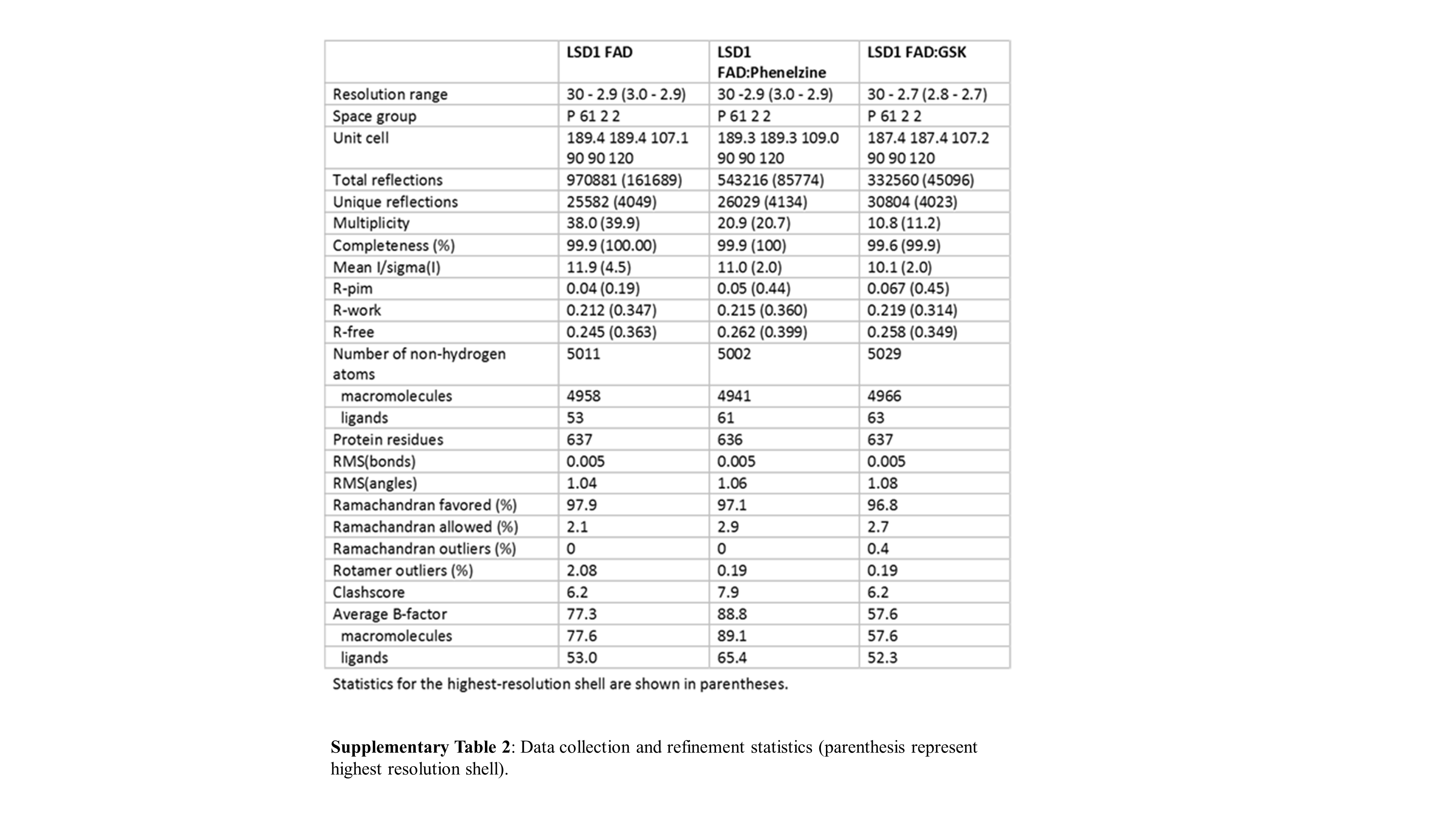

Supplement: Supplementary file 4 [file Image_4.TIF]
